# The role of the odorant receptors in the formation of the sensory map

**DOI:** 10.1186/s12915-021-01116-y

**Published:** 2021-08-27

**Authors:** Simona Francia, Claudia Lodovichi

**Affiliations:** 1 Center for Synaptic Neuroscience and Technology, Istituto Italiano di Tecnologia, Genoa, Italy; 2 Veneto Institute of Molecular Medicine, Padua, Italy; 3 Neuroscience Institute CNR, Via Orus 2, 35129 Padua, Italy; 4 Department of Biomedical Sciences, University of Padua, Padua, Italy; 5 Padova Neuroscience Center, Padua, Italy

## Abstract

In the olfactory system, odorant receptors (ORs) expressed at the cell membrane of olfactory sensory neurons detect odorants and direct sensory axons toward precise target locations in the brain, reflected in the presence of olfactory sensory maps. This dual role of ORs is corroborated by their subcellular expression both in cilia, where they bind odorants, and at axon terminals, a location suitable for axon guidance cues. Here, we provide an overview and discuss previous work on the role of ORs in establishing the topographic organization of the olfactory system and recent findings on the mechanisms of activation and function of axonal ORs.

## Introduction

**Fig. 1:**
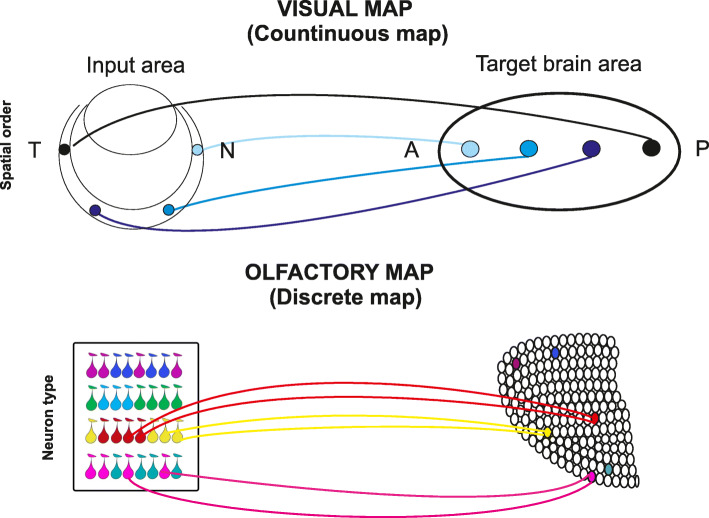
Continuous and discrete sensory maps. Top. Schematic diagram of a continuous sensory map in the visual system. The spatial distribution of sensory information in the input area is maintained in the target area. In this organizational plan nearby neurons in the input area form synapses with nearby neurons in the target area. T = temporal, N = nasal, A = anterior, P = posterior. Bottom. Schema of a discrete map in the olfactory system. The topographic organization of the target area reflects the type and not the spatial distribution of sensory inputs. Olfactory sensory neurons expressing the same odorant receptor (indicated by drop shapes with the same color, bottom left) converge to form glomeruli (color filled circles) in specific locations of the target area (bottom right)

In mammals, sensory pathways begin in the peripheral sensory neurons where sensory stimuli are translated in electrical signals. These electrical inputs are then transferred to higher brain areas along specific neuronal circuits to provide an internal representation of the external world. The spatial segregation of sensory afferents provides a topographic map that encodes the quality, the intensity, and the location of sensori stimuli. Two different types of neuronal maps have been described: continuous and discrete (Fig. 1) [1–4].

In continuous neuronal maps, nearby neurons in the periphery project to nearby neurons in the target brain area, thus preserving the spatial pattern. In most sensory modalities, sensory neurons in the sense organs are spatially ordered according to the physical features of the stimulus they detect. This spatial and sensory arrangement is maintained in higher brain areas resulting in continuous neuronal maps. The visual map is considered the prototypic example of a continuous topographic map, where the spatial segregation of retinal ganglion cell (RGC) axons into the target reflects the spatial relation among RGC in the periphery (Fig. 1, top) [2].

In discrete neuronal maps, the spatial organization of the target area reflects discrete features rather than the spatial arrangement of neurons in the peripheral sense organs. The topographic organization of the olfactory bulb is a typical example of a discrete neuronal map, where the discrete feature on which the map is formed is represented by the odorant receptor type. In olfaction, in the peripheral sheet, i.e., the olfactory epithelium, sensory neurons which express a given odorant receptor (OR), and therefore process-specific sensory information, are not distributed according to a distinct spatial order but exhibit a coarse topographic organization. Spatial relation among receptor neurons cannot, therefore, instruct the segregation of sensory afferents to the target. In this scenario, a different strategy has been exploited to build a topographic map. Olfactory sensory neurons (OSNs) expressing the same OR project in specific loci of the olfactory bulb (glomeruli) to form synapses with the postsynaptic cells resulting in a neuronal map with discrete information. The projections of sensory axons pivot on the identity (i.e., type) of the odorant receptors which governs the formation of a discrete sensory map (Fig. 1 bottom) [3, 5, 6].

In this review, we provide an overview of the steps that unraveled the OR identity as the discrete feature upon which the olfactory map is built and discuss new insights on the role of the axonal ORs in the topography of the olfactory bulb (OB) in rodents (rats and mice).

### The olfactory system

The olfactory system (OS) is a very ancient and evolutionary highly conserved system from flies to mammals. It possesses an extraordinary discriminatory power, able to detect and discriminate thousands of different odors present in the environment even at very low concentrations. In terrestrial mammals, including rodents, odorant molecules (odorants) are typically volatile, small (generally with a molecular weight below 400 Da), and predominantly organic molecules dissolved in the air that enters the nasal cavity. Odorants encompass a wide variety of organic compounds that differ for carbon chain length, charge, shape, and functional group(s). Based on the functional group, odorants are classified into different classes such as aldehydes, ketones, alcohols, carboxylic acids, amines, esters, thiols, and nitriles. In aquatic environments, odorant molecules are mostly amino acids [7]. How the percept of an odorant is achieved remains largely to be understood, although it is known that the structure, namely the length of the carbon chain and the functional group of the odorant molecules, has a role in defining the percept. In addition, the presence of individual variability in detecting odorants, such that the same organic molecule can elicit different percepts in different individuals, is well established [8–11]*.*

Odorants are sensed by the OSNs located in the main olfactory epithelium (MOE), which lays the posterior part of the nasal cavity. OSNs are bipolar cells with a single apical dendrite and a thin unmyelinated and unbranched axon that projects directly to the OB, a part of the forebrain consisting of two bilateral structures above the nasal cavity, where olfactory information is first processed. The OSN apical dendrite ends in a swelling knob-like structure from which several thin and long cilia protrude in the mucus of the nasal cavity [12], where they are exposed to the incoming air and odorants. ORs are expressed at the cilia where they bind odorants and trigger the chemo-electric signal transduction.

### The odorant receptors

The large family of ORs was discovered more than 30 years ago by L. Buck and R. Axel (1991) in a seminal work where they cloned and characterized a subgroup of a multigene family of G protein-coupled receptors (GPCRs), whose expression was limited to the MOE [13]*.* The OR sequences have been then identified in different invertebrates (nematode and fruit fly) [14–17] and vertebrates (amphibians, lizards, fish, birds, and mammals) [18–23]. In vertebrates, OR genes are phylogenetically classified based on their sequences homology in: Class I (vertebrate marine heritage) activated by water-soluble ligands (i.e., aldehydes, alcohols, amino acids, and aliphatic acids) and Class II (mammalian terrestrial heritage) binding airborne (volatile) ligands [24]. In mammals, the OR repertoire encompasses ~1000 OR sequences harbored in clusters in almost all chromosomes, resulting in one of the largest gene family. In rodents, 20% of OR genes are pseudogenes (i.e., non-functional sequences of DNA that resemble functional genes), while in humans, the percentage of OR pseudogenes is significantly higher, reaching 60% [25, 26]. These differences in OR pseudogenes likely reflect the beahvioral and ecological differences of the OS among species.

#### OR structure

The ORs belong to the large family of GPCRs, a class of receptors that mediate a vast range of important physiological functions, such as responses to hormones, neurotransmitters, and sensory stimuli (being involved in the transduction of visual and chemical stimuli) (Rosenbaum DM 2009). ORs share several common features with GPCRs, such as the coding region that lacks introns and the basic structure characterized by 7 transmembrane alfa helical domains, separated by intracellular and extracellular loops, with various conserved regions, and connected to an extracellular N-terminus and intracellular C-terminus [22, 27–29]. Among the OR sequences, the range of homology varies from 40 to over 90%. Most interesting are the regions of hypervariability, which are likely to represent the binding site for odorants. To this end, functional studies based on site directed mutagenesis and ligand docking simulation seem to indicate the transmembrane (TM) domains 3, TM5, TM6, and TM7 as putative regions of binding [30–33]. The binding site of ORs to the coupled G-protein is a conserved tripeptide motif, aspartate-arginine-tyrosine (DRY), located at the cytoplasmic side of the transmembrane domain III [29, 33]. Mutations in the DRY motif hampers the coupling of OR to G-proteins, abolishing cyclic adenosine monophosphate (cAMP) rise and response to odorants [34]. On the other hand, mutations of the serine in KAFSTC, a highly conserved motif among ORs members, result in a significant increase in odorant responsiveness, suggesting that KAFSTC is involved in the conformational change of the receptors that modulates G-protein coupling efficacy [32]. Although several studies based on computational approaches, docking interaction simulations, structural models, and targeted mutagenesis have been employed to predict the structure-function relation of the ORs [32, 35–37], our understanding of the impact of the OR conformation on its functionality remains limited in the absence of the crystal structure of the ORs.

In this scenario, the crystal structure of other GPCRs, in particular the β-adrenergic receptor (β-AR), represented a breakthrough in understanding how the structure of the GPCRs defines their functionality and provided some insights to decipher, at least in part, the structure-function relation also for ORs [38, 39]. The β-AR receptors associate with G_s_ and adenylyl cyclase (AC) to form the β-AR complex, leading to an AC-dependent increase in cAMP and activation of protein kinase A (PKA), which, in turn, promotes phosphorylation of several intracellular targets. Being members of the GPCR family, ORs are coupled to a similar intracellular signaling pathway (see below). GPCR were, at first, depicted as bimodal receptors, which switch between inactive and active state. Subsequent studies unraveled that GPCR, in particular the β-ARs (among the best characterized GPCR), are dynamic proteins that can assume different conformations associated to distinct levels of activity, hence different biological responses. The effect of the ligand on the structural and biophysical properties of the receptor, which in turn determine the biological responses, is known as ligand efficacy. Natural and synthetic ligands are divided in full agonists fully activate the receptors, partial agonist provide submaximal activation, while inverse agonist prevent the receptor activation. GPCRs exhibit also a considerable amount of agonist-independent activity, indicated as constitutive or basal activity [38, 40]. In a similar way, ligands of the OR can act as agonists that fully activate the receptor, or antagonists that block the response, or partial agonists that elicit partial activation [8, 41–44]. Furthermore, also the ORs exhibit ligand-independent activation, which drives the spontaneous firing (i.e., basal activity) of OSNs. Evidence of OR spontaneous activation comes from OSNs expressing inactive mutant ORs, which completely lack spontaneous activity. In this context, it has to be noticed that although OSN basal activity is determined by the ORs, even OSNs expressing the same OR exhibit a substantial variation in their firing rate [45, 46]*.* The spontaneous activity of the ORs was shown to have a role in the formation of the glomerular maps (see below). The similarities between the structure and the signaling pathway of OR and β-AR inspired challenging experiments aimed to pinpoint the role of the ORs in the development of the glomerular map [47, 48].

#### The OR signaling pathway

**Fig. 2:**
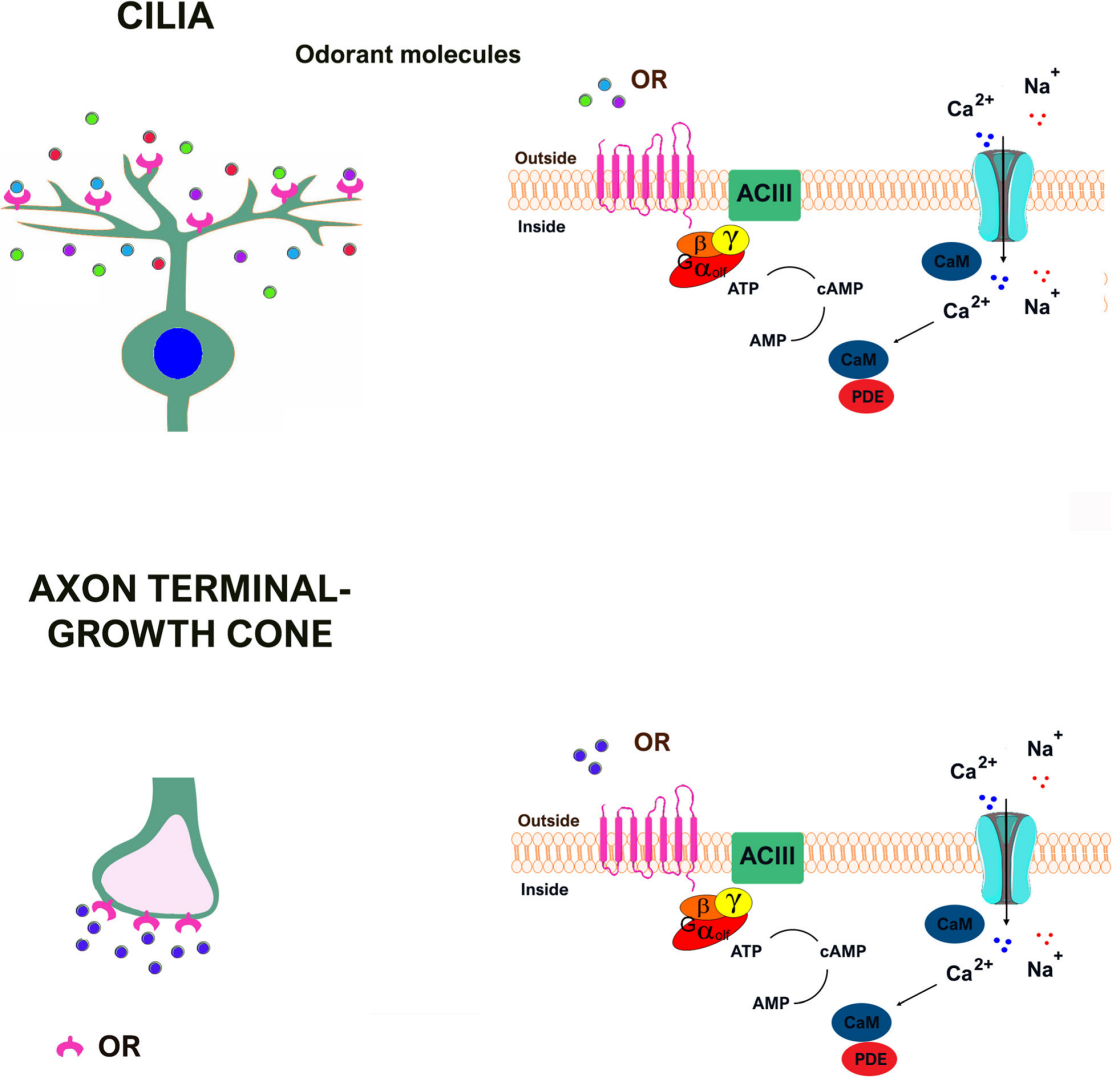
Local increase of cAMP and Ca^2+^ coupled to odorant receptors expressed at the cilia and at the axon terminal. Schematic diagram of the signaling pathway coupled to the odorant receptors (OR) expressed at the cilia and at the axon terminal of olfactory sensory neurons. Both the ORs expressed at the cilia and the ORs at the axon terminal are coupled to local increase of cAMP and Ca^2+^. At the cilia, the influx of Ca^2+^ through the CNG channels leads to the opening of Ca^2+^-dependent Cl^-^ channels (not shown in figure). The presence of these Cl^-^ channels also at the axon terminal remains elusive. AC III adenylyl cyclase III, CaM Calmodulin, PDE phosphodiesterase, PEBP1 phosphatidylethanolamine binding protein-1 indicated by blue dots, at the axon terminal, while odorant molecules are indicated by dots of different colors, at the cilia

The signaling cascade triggered by odorants is well characterized. Upon odorant exposure, OR activates G_olf_, a specific G-protein that stimulates adenylyl cyclase type III to synthesize cAMP. cAMP in turn opens the olfactory specific nucleotide-gated (CNG) channels leading to an influx of Ca^2+^ and Na^+^ inside the cell (Fig. 2) [49–51]. The increase of Ca^2+^ level induces the opening of Ca^2+^-activated Cl^-^ channels, allowing an outflow of Cl^-^ which further depolarizes the neuron to generate action potentials. Upon odorant binding, the increased level of Ca^2+^ at the cilia mediates not only the activation but also the desensitization of OSNs. Ca^2+^ has important negative feedback effects on different steps of the intracellular signaling cascade, including the CNG channels, adenylyl cyclase, and phosphodiesterases [52, 53].

Each OSN expresses only one type of OR in a repertoire of more than 1000 OR genes. The mutually exclusive and monoallelic expression of the OR gene is thought to provide a mechanism that assures the expression of a single type of OR in each OSN [54]. In this way, the expressed OR defines the molecular receptive range and the functional identity of each OSN. As a result of this genetic pattern of OR expression comes the rule: “one OSN-one OR”. It is therefore critical that once a given receptor has been chosen for expression, this translation choice is maintained for the entire life of the cell, which has to preserve its identity and its specific connections with the postsynaptic targets. To this end, several studies have focused on the stability of the OR expression. What has been found is that immature OSNs, before forming synapses with the post-synaptic cells, can switch OR expression choice, although with very low probability. In contrast, OSNs expressing a mutant OR switch OR expression with a high probability to reach the situation where only a single functional receptor is expressed. This mechanism suggests that the expression of a functional OR elicits a feedback mechanism that ends switching [55–57].

The presence of a repertoire of more than 1000 ORs enables the identification of a wide range of odorants, further amplified by the distinct nature of the OR–odorant interaction. By performing calcium imaging dynamics as a readout of OR activation in combination with single RT-PCR, Malnic and coauthors (1998) [9] demonstrated that each OR can recognize several odorants and, in turn, each odorant can bind and activate several ORs. This pattern of OR–ligand interaction has been defined “combinatorial code.” Moreover, they found that the identity of different odorants is encoded by distinct combinations of activated ORs. However, each OR can serve as a component of several combinatorial patterns of ORs. Given the extraordinarily high number of possible combinations of ORs, the combinatorial code allows for the discrimination of an almost unlimited number of different odorants [9, 58].

#### Deorphaning ORs

For a long time, it has been difficult to correlate a given OR to its specific cognate ligands. The biggest obstacle in this process was the impossibility to express functional ORs in heterologous systems, because of the OR retention in the endoplasmic reticulum and subsequent OR degradation in the proteasome [59, 60]. Firestein group overcame this limitation using the olfactory epithelium as an expression system and exploited adenovirus vectors expressing I7-OR to drive the expression of I7-OR in a large population of OSNs. To unravel the ligands of I7-OR, the response to a wide panel of odorants was tested by recording the electro-olfactogram in infected and non-infected MOE. The electrophysiological responses indicated that I7-OR infected epithelium exhibited higher responses to a few molecules [61]. This approach allowed the identification of the first ligands of a given OR but resulted laborious and not suitable for large-scale screening.

A breakthrough in de-orphaning ORs was achieved by Matsunami and collaborators [62, 63]. They screened for genes inducing cell membrane expression of ORs in heterologous system (i.e., HEK and Hana cells). They identified accessory proteins indicated as Receptor Transporting Protein (RTP) 1 (RTP1) and 2 (RTP2). These proteins were expressed specifically in OSNs and they were found to favor the targeting of the ORs to the cell membrane, to interact with ORs, and enhance odorant-response of the ORs expressed in heterologous systems [62, 64]. To unravel the ligands of single ORs expressed in heterologous system, RTPs were co-transfected with the OR sequence and a firefly luciferase reporter gene under the control of a cAMP response element (CRE). By performing luciferase assay, the luciferase production was measured as a function of OR activation in vitro in response to subsets of odorant molecules. This approach allowed de-orphaning a large number of ORs [62, 63]. It is worth noticing that in this approach, odorant OR activation is inferred from the activation of the OR intracellular signaling pathway (i.e., cAMP). Indeed, up until now, no reliable assays to ascertain the direct binding of ORs with their ligand have been devised, although a binding assay for other G-protein receptors has been developed, such as TANGO [65]. This approach enables monitoring the GPCR activation with high sensibility and selectivity without hampering the endogenous pathway. This strategy was developed for three different receptor classes: the tyrosine kinase, GPCRs, and steroid hormone receptors, leaving OR still without a specific binding assay [65].

A step forward in the identification of the OR–ligand interaction has been recently achieved in *Drosophila* as Butterwick and collaborators [66] presented a cryogenic electron microscopy (Cryo-EM) structure of the insect OR Orco. Though insect ORs are unrelated to GPCR, this discovery could prompt new experimental approaches to decipher the structure of mammalian ORs. Therefore, in the absence of reliable binding assays for mammalian ORs, odorants have been identified as ligands of a given OR on the basis of the activation of the OR intracellular signaling pathway. In this scenario, the increase of the OR-derived cAMP and/or Ca ^2+^ is considered the signature of OR activation. In line with this approach, the identification of ligands for the axonal ORs also exploited the functional activation of the intracellular pathway coupled to the ORs (i.e., local increase of cAMP and Ca^2+^) as readout of OR activation ( [67] and see below).

### The topography of the olfactory system

In rodents, the MOE exhibits a coarse topographic organization. Sensory neurons expressing the same OR are located within large but confined areas along the dorso-ventral axis of the MOE. Within the same zone, however, OSNs expressing different ORs are intermixed [68, 69]. Subsequent studies indicated that the OR expression is continuous and overlapping in distinct zones across the dorso-medial to the ventro-lateral axis of the MOE [70–72]. The mechanism underlying the restriction of OR gene expression within a given zone remains largely elusive.

**Fig. 3:**
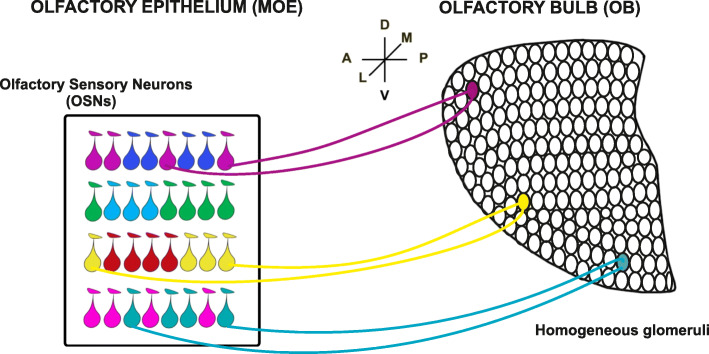
Projections of olfactory sensory neurons (OSN) to the olfactory bulb (OB). OSNs expressing a given odorant receptor (OR, indicated by drop shapes with the same color) are confined in one of the partially overlapping zones in which the olfactory epithelium has been subdivided along the dorso-ventral (D-V) axis. Within each zone, OSNs expressing different ORs (indicated by drop shapes of different colors) are intermingled. OSNs expressing the same type of ORs project to form glomeruli (filled circle of the same color of the corresponding OSNs) in specific loci of the olfactory bulb (OB). There is a correspondence between the zonal organization of the epithelium along the Dorso-Ventral (D-V) axis and the projection of OSNs along the D-V axis of the OB. OSNs harbored in the most dorsal zone of the epithelium project to the most dorsal area of the bulb, whereas OSNs located in the most ventral zone of the epithelium, project to the ventral area of the bulb. A = anterior, P = posterior, M = medial, L = lateral, D = dorsal, and V = ventral

**Fig. 4:**
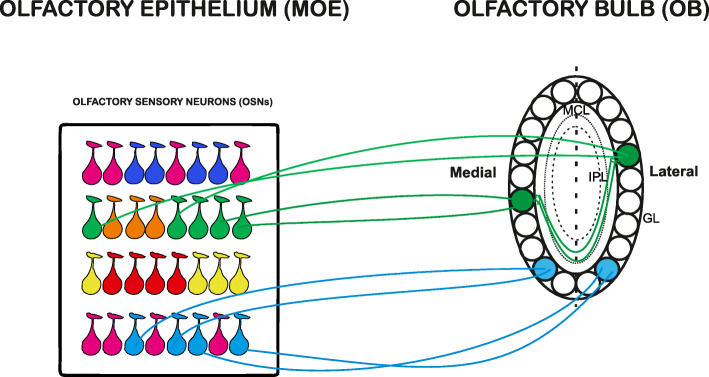
Homologous glomeruli within the olfactory bulb (OB). Olfactory sensory neurons (OSNs) expressing the same odorant receptor (OR, depicted as drop shapes with the same color) form one glomerulus on the medial side and one glomerulus on the lateral side of each OB. This couple of glomeruli is indicated as homologous or isofunctional glomeruli because they both processed information related to the same OR. The homologous glomeruli are connected through a link related to external tufted cell (ETC) projections (indicated with green lines connecting the homologous glomeruli, green filled circles), which terminate in the internal plexiform layer (IPL), just underneath the homologous glomerulus on the opposite side of the bulb (depicted by the green lines ending just beneath the homologous glomeruli). This connection is reciprocal. For simplicity, only a few layers of the OB are indicated. GL = glomerular layer, MCL = mitral cell layer, IPL = internal plexiform layer

An orderly spatial distribution of sensory afferents is achieved in the OB. Here, axons of OSNs bearing the same OR converge to form synapses with the postsynaptic cells in precise loci (i.e., glomeruli) on the lateral and the medial side of each OB, respectively, in each animal (Figs. 3 and 4) [73, 74]*.* The convergence of like-axons to form glomeruli in the OB was directly visualized thanks to an elegant approach of targeted mutagenesis in a genetically modified line of mice, P2-IRES-Tau-LacZ mice. In this line of mice, the endogenous P2-OR was co-expressed with a reporter gene, Tau-lacZ, which allowed to easily identify P2 neurons in the MOE and the corresponding glomeruli in the OB [75]. In subsequent studies, the replacement of tau LacZ with a green fluorescent protein (GFP) enabled the direct visualization of OSNs and the corresponding glomeruli [76]. Therefore, the rule “one sensory neuron-one receptor” extends to the glomeruli, which follow the law “one glomerulus - one receptor”. As a result of this organization, a hallmark of mature glomeruli is that they are formed exclusively by fibers expressing the same OR, indicated as homogenous glomeruli [75, 77].

**Fig. 5:**
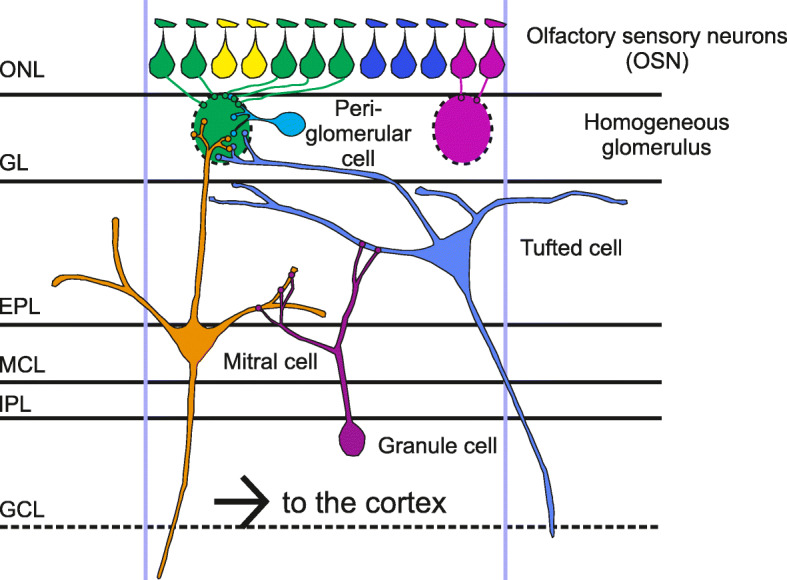
Neuronal circuitry in the olfactory bulb (OB). Schematic diagram of the connectivity between pre and postsynaptic cells in the OB. Within each glomerulus, olfactory sensory neurons (OSNs) expressing the same odorant receptor (OR) (indicated by drop shapes with the same color) form synapses with the postsynaptic cells, namely the mitral cells (MC) and tufted cells (TC), along with the periglomerular cells. The MC and TC extend their single apical dendrite within a single glomerulus whereas the lateral dendrites run along the external plexiform layer and form dendro-dendritic synapses with the granule cells, the major GABAergic inhibitory interneurons of the OB. The glomerulus defines, therefore, a functional unit, known also as olfactory column, where sensory information related to a give OR is processed. ONL = olfactory nerve layer, GL = glomerular layer, EPL = external plexiform layer, MC = mitral cell layer, IPL = internal plexiform layer, GCL = granule cell layer

Glomeruli are spherical structures of neuropil where OSNs expressing a specific OR form synapses with the post-synaptic cells of the OB, namely mitral and tufted cells (MC and TC), along with periglomerular cells. MC and TC elongate their single apical dendrite exclusively in a given glomerulus. As a result of this pattern of connectivity, a glomerulus defines a functional unit that processes sensory information related to the OR expressed by the OSNs forming the glomerulus itself (Fig. 5). In analogy with orientation and ocular dominance columns of the visual system, these functional units are indicated also as “olfactory columns” [12]. This spatial segregation of the sensory afferents provides the topographic map of the OB which encodes the quality and intensity of odorant stimuli [3, 6, 27, 78]. According to the architecture of the topographic map and the nature of the combinatorial code (see above), an odorant is encoded by a spatial pattern of activated glomeruli, as demonstrated by functional imaging experiments [79–84]. Therefore, the specific location and organization of glomeruli within the OB map are essential for proper odorant coding.

The converge of axons expressing the same OR to form a glomerulus on the medial and a glomerulus on the lateral side of each bulb results in two mirror-symmetric maps of isofunctional glomeruli, also known as homologous glomeruli (Fig. 4). Compelling evidence revealed that these two maps are two halves of an integrated map [85], where isofunctional glomeruli are reciprocally linked by an inhibitory projection related to external tufted cells (ETC) (Fig. 4). Namely, ETC connected to a given lateral glomerulus project their axons to a restricted region in the internal plexiform layer just underneath the medial homologous glomerulus, i.e., the glomerulus formed by axons expressing the same OR, located on the opposite side of the bulb. The ETC axons form excitatory synapses with the granule cells connected to the medial homologous glomerulus. These granule cells, in turn, make inhibitory synapses with the ETC connected to the medial homologous glomerulus. This connection is reciprocal (Fig. 4) (Belluscio, 2002; Lodovichi 2003). This intrabulbar link is present in both bulbs of the same animal and in bulbs of different animals, providing the second level of topography in the OB.

From the pattern of neuronal wiring emerges that the topographic organization of the OB hinges on the OR identity which defines the molecular receptive range of the OSNs and represents the “discrete feature” upon which the olfactory map is built, being glomeruli functional units that process sensory information related to a given OR.

### Mechanisms underlying the formation of the sensory map: the role of the ORs

The role of the OR in the formation of the sensory map was unraveled by a set of elegant genetic experiments, whose results demonstrated that alterations of the OR sequence led to the altered convergence of sensory neurons and the disruption of the sensory map [86]. In the Axel lab, Wang found that the deletion or non-sense mutations of the P2-OR gene prevented the convergence into glomeruli of these axons, which instead targeted different locations and appeared as wandering fibers in the OB [86]. In subsequent experiments, it was demonstrated that the deletion of the OR coding sequence leads OSNs to express a different OR and consequentially target other glomeruli with respect to the original one [55–57]. This evidence corroborated the relation between the OR identity and the specific glomerular loci within the OB.

Wang (1998) then performed a series of substitution (i.e., swapped) experiments, where a given receptor sequence, such as P2-OR, was replaced with another OR sequence, such as the one of the M71-OR. The axons expressing the swapped OR, M71>P2, converge to form a glomerulus in a different location with respect to the site of the original P2 or M71 glomeruli. Wang and collaborators found that even when they swapped OR gene sequence with a high degree of homology, located within the same gene-cluster and expressed by OSNs localized in the same MOE zone, such as P2-OR and P3-OR genes, the P2-P3 expressing axons converge to form glomeruli in locations different from the positions of the original P2 or P3 glomeruli [86]*.* These results indicated that the location of the “swapped” glomerulus, in respect to the location of the original glomeruli, is affected by several factors such as 1. the degree of homology between the swapped OR sequences, 2. the position in the olfactory epithelium zones of the OSN expressing the 2 swapped receptors, and 3. the chromosomal location of the OR swapped coding regions. The information obtained by the substitution experiments indicates that the OR plays an instructive role in the convergence of OSN axons, although it is not the only determinant.

Another set of swapping experiments performed in Mombaerts laboratory reached similar conclusions and added new information. Through several substitution experiments between M71 and M72-ORs, they identified the “core” sequence within the OR gene that defines the OR identity and dictates the target of the axonal projection. They also found that the level of OR expression was important for normal OSN convergence [87]. Due to the similarities of the ORs with other members of the GPCR family (see above), they replaced the OR sequence with the sequence of other GPCR [47], obtaining very different results according to the type of GPCR swapped. In a set of experiments, they replaced the sequence of the β-AR in the locus of M71 OR. Analyzing the convergence of β-AR>M71, they found that the swapped axons coalesce to form glomeruli in the OB, although in different locations with respect to the original M71 glomeruli. In contrast, swapping the vomeronasal receptor V1R, namely the V1rb2 [88] in the M71 locus, exerted a very different effect. OSNs expressing V1rb2 did not converge to form glomeruli in the OB [47]. The different outcome of these substitution experiments is likely due to the different intracellular signaling cascade coupled to β-AR in respect to V1rb2. Both the OR and β-AR are coupled to cAMP (see above), which is known to play a key role in the coalescence of like-axons [34, 89–92]. In contrast, V1rb2 is a GPCR not coupled to cAMP. These results suggest that the OR-derived cAMP regulates the coalescence of like axons to form glomeruli, while the OR is required for targeting OSN axons in specific loci in the OB.

In these studies, it emerged that the OR dictates the glomerular target; in other words, it could act as an axon guidance molecule. If the OR has to govern axon targeting, it has to be expressed on the axon terminal, a suitable location for a putative axon guidance molecule [86]. This hypothesis was corroborated by experiments which revealed the ORs are expressed on the distal portion of the axons, whereas the proximal segment of the axon is devoid of OR expression [93, 94]. Furthermore, it was shown that the ORs are also locally translated at the axon terminal-growth cone [95]. The specific and exclusive expression of the ORs at the cilia and at the axon terminal-growth cone of OSNs suggested a specific function of the ORs in these two locations. However, the mere expression of the ORs at the axon terminal without knowing whether the ORs in this location are functional and if yes, which is the signaling pathway coupled to them, prevented defining the role of the axonal ORs.

### Intracellular signaling pathway associated with the axonal ORs

The first evidence that the axonal ORs are functional was provided by Maritan and collaborators [96]. By imaging the spatio-temporal dynamics of cAMP and Ca^2+^ in isolated OSNs, they found that odorant application at the axon terminal of OSNs resulted in a localized increase of cAMP and influx of Ca^2+^ through cyclic nucleotide-gated channels (CNG) (Fig. 1) (Maritan et al., 2009). The response to odorants of the axonal ORs demonstrated that the ORs at the two opposite poles of OSNs are coupled to local increase of cAMP and Ca^2+^, although it is unlikely that odorants are the natural ligands of the axonal ORs. In Maritan et al. (2009), odorants were employed with the exclusive goal to unravel the functionality and the signaling pathway coupled to the axonal ORs, as no other ligands were known at the time. Upon odorant application at the axon terminal, the local increase of cAMP was followed by the activation and translocation of protein kinase A (PKA) in the nucleus [96, 97]. These findings suggest that Ca^2+^ and cAMP coupled to the axonal ORs can exert their action in two distinct sites: 1. locally, regulating cytoskeleton dynamics to modulate elongation and turning of the axon terminal-growth cone and 2. at the nucleus, via PKA activation, to regulate the expression of molecules involved in axon guidance process [98].

A consistent body of evidence indicates that in OSN odorant-induced activation of ORs is coupled also to the synthesis of another second cyclic messenger, cyclic guanosine monophosphate (cGMP). The long and sustained rise of cGMP suggested that this second messenger is not involved in the detection of odorants but rather in long cellular responses, including axon elongation and targeting [99–101]. Real-time imaging experiments in isolated OSNs demonstrated that cGMP is locally produced both at the cilia and at the axon terminal. Furthermore, it was reported that there is a strict interplay between cAMP, cGMP, and Ca^2+^ [102]. Noteworthy, similarly to cAMP, also cGMP can exert its action locally, at the growth cone, and at the nuclear level, via PKA translocation and CREB activation [102]. The local synthesis of cAMP, Ca^2+^, and cGMP coupled to the axonal ORs is of relevance for the putative role of the ORs as axon guidance cues, as these second messengers are known to play a key role in axon elongation and turning in several systems [103–105], including the olfactory system [34, 89]*.* In addition, the local expression of functional ORs at the OSN axon terminal–growth cone is in line with a substantial body of evidence indicating that the axon terminal–growth cone acts as an autonomous compartment. Indeed, it is characterized by complex machinery able to translate locally the mRNA of axon guidance cues, endowing the axon terminal with the ability to respond promptly to cues encountered on the way towards its target [106].

To ascertain which second messenger coupled to the ORs could have a critical role in the convergence of OSN axons, Imai and collaborators (2006) generated a mutant defective receptor, introducing a mutation in the conserved tripeptide motif, aspartate-arginine-tyrosine (DRY), at the cytoplasmic end of the III transmembrane domain of the OR. This domain is required for the coupling of ORs to G-proteins; therefore, as a consequence of this genetic mutation, cAMP synthesis was abolished. Axons carrying the DRY mutation never entered the glomerular layer and remained in the olfactory nerve layer [34]. To seek to understand how the OR-derived cAMP directs OSN axons to their target, they searched for molecules whose expression could be regulated by cAMP. Using microarrays and RT-PCR, they identified Neuropilin1, whose expression was high in OSNs with a high level of cAMP, while it was missing in DRY-mutant OSNs [34]. Neuropilin1 was known to modulate glomerular location along the A-P axis, since mutations in Neuropilin1 or Semaphorin3A (Sema3A)—its repulsive ligand, disrupted the arrangement of glomeruli along the A-P axis [107–109]. These results highlighted the crucial role of the OR-derived cAMP in defining the location of glomeruli along the A-P axis of the neuronal map in the OB.

Altogether, these data provided important insights on the role of the OR in the formation of the sensory map, but they leave open critical questions. The DRY-motif mutant OR (Imai et al., 2006, see above) prevented to understand which OR is involved in directing OSNs to their target, since the genetic manipulation involved both the ORs expressed at the cilia and the ones at the axon terminal. Furthermore, the mechanism that triggers the increase of the OR-derived cAMP remained to be determined.

### Identification of the first putative ligand of the axonal OR: phosphatidylethanolamine binding protein-1 (PEBP1)

In agreement with previous speculations [86], Zamparo and colleagues (2019) [67] postulated that the axonal OR could act as an axon guidance cue, being activated by molecules expressed in the OB. Through an unbiased screen of OB molecules, Zamparo et al. identified a pool of molecules expressed in the OB that, applied at the axon terminal, were able to elicit Ca^2+^ rise, locally. To ascertain that this Ca^2+^ response was due to the activation of the axonal ORs, Zamparo exploited the approach developed by Matsunami (see above and Saito et al., 2004) to express distinct ORs in HEK cells. In this contest, a prompt Ca^2+^ rise in response to the active pool of OB molecules and to the specific odorants was observed only in HEK cells expressing ORs but not in HEK cells devoid of OR expression. These data suggested that the OB molecules were able to activate the axonal ORs. The functional outcome of these findings was deduced with an in vitro turning assay [103]. Application of gradient of molecules (including the OB cues) able to regulate cAMP and Ca^2+^ was capable of modulating the elongation and steering of isolated OSN axons. Among the pool of active molecules, phospatidylethanolamine binding protein 1 (PEBP1) was identified, by mass spectrometry, as the first putative ligand of the axonal ORs [67]. PEBP1 is a small molecule (about 21 kDa), with unknown function, expressed in neurons and non-neuronal cells in many brain areas [110]*.* It can be secreted, although through a non-canonical pathway and its receptor remains elusive [111]. PEBP1 is the precursor of an undecapeptide named hippocampal cholinergic neuro-stimulating peptide (HCNP) located at its N-terminal [112]. HCNP was isolated from rat hippocampus and is expressed in several regions of the central nervous system [113, 114]. HCNP is involved in the differentiation of cholinergic neurons in the medial septal nuclei both in vitro and in vivo, hence its name, hippocampal cholinergic neuro-stimulating peptide [112].

In mouse and rat OB, PEBP1 is expressed mostly in periglomerular cells, a suitable location for a molecule supposed to drive incoming sensory axons. Furthermore, PEBP1 exhibits a general antero-posterior gradient. However, locally, PEBP1 has a patchy distribution, where glomeruli with low or high levels of PEBP1 expression are intermixed [67]. This pattern reflects the discrete nature of the olfactory map and, indeed, other axon guidance molecules, such as Neuropilin1, exhibit a similar pattern of general/local expression [115]. This expression profile strikingly differs from the continuous spatial distribution of axon guidance cues found in the continuous map, such as the visual map [2].

**Fig. 6:**
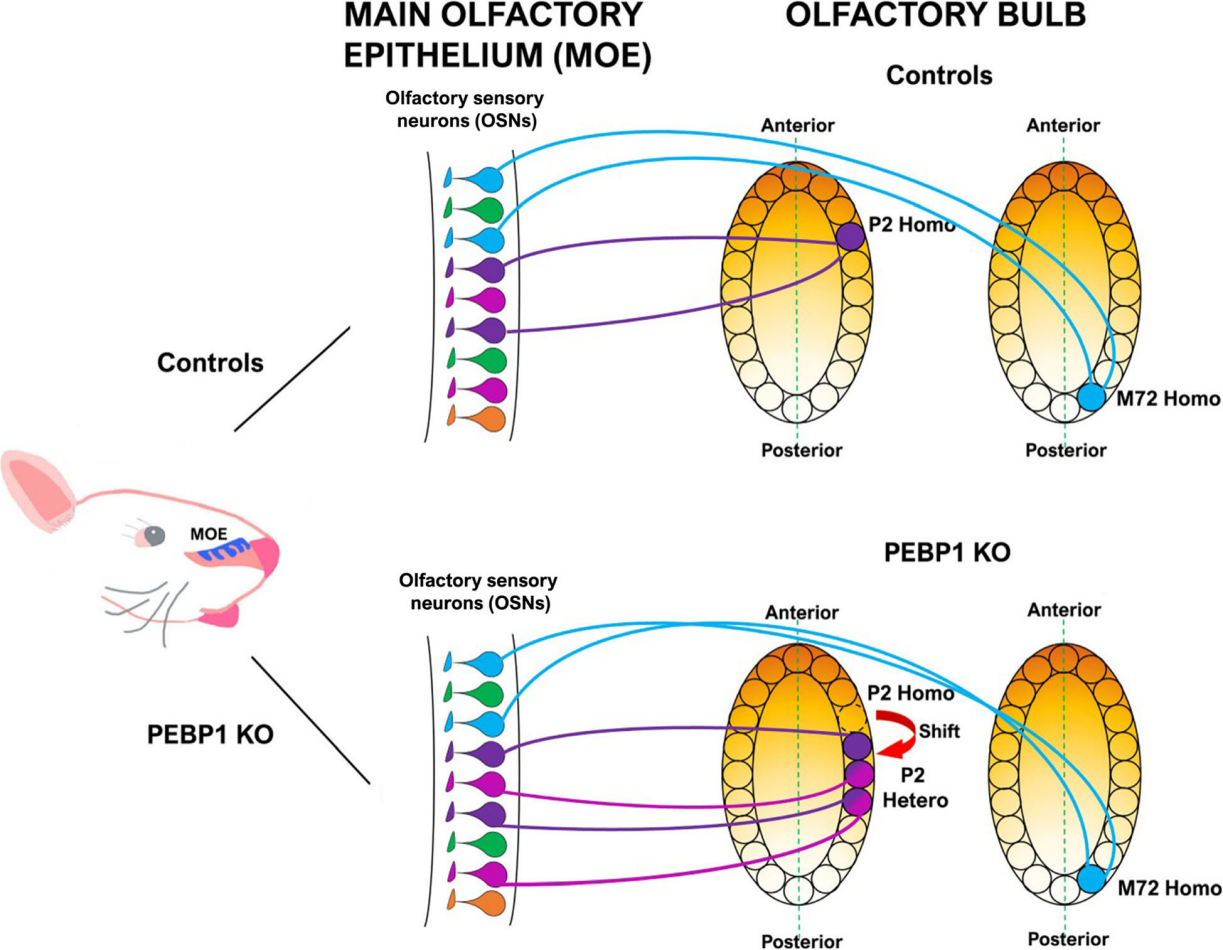
Altered convergence of P2-expressing sensory axons in the olfactory bulb (OB), in the absence of phosphatidylethanolamine binding protein-1 (PEBP1). Schematic diagram of the convergence of olfactory sensory neurons (indicated by drop shapes of different colors) expressing P2 (purple) and M72 (light blue) odorant receptors to form glomeruli (purple and light blue filled circles, respectively) in the OB in control (top) and PEBP1 KO (bottom) mice. Top, in controls, P2-OR and M72-OR expressing axons converge to form homogeneous glomeruli (homo P2 and homo M72) in specific loci of the OB. In PEBP1 KO mice (bottom), the location of the main homogeneous P2 glomerulus (P2 homo, purple filled circle) is shifted (red arrow) along the antero-posterior axis, in respect to controls (original location is indicated by an empty dashed circle). Furthermore, in PEBP1 KO mice, P2 axons target additional glomeruli, which are formed by fibers expressing different odorant receptors (i.e., heterogeneous glomeruli (P2 hetero), indicated by filled circle with two colors, purple and pink). The convergence of M72 expressing axons is similar in controls (top) and mutant mice (bottom). These results indicate that PEBP1 acts as a putative ligand for P2-axonal receptors and not for M72-axonal receptors. In the schematic sections of the olfactory bulb, shaded yellow backgrounds indicate the general antero-posterior gradient of PEBP1. MOE = main olfactory epithelium

PEBP1 elicited Ca^2+^ increase at the axon terminal of OSNs and in HEK cells expressing specific ORs. Among the OR tested, all but one, i.e., M72-OR, showed Ca^2+^ response upon PEBP1 stimulation, suggesting that other putative ligands of axonal ORs remain to be identified. The physiological role of PEBP1 was demonstrated in vivo by studying the olfactory map in mice carrying a null mutation for PEBP1. In this transgenic line of mice, P2-OR expressing axons targeted the main glomerulus but also several additional heterogeneous glomeruli, i.e., glomeruli formed by fibers expressing different types of ORs. Furthermore, the main P2 glomerulus was shifted along the A-P axis with respect to controls [67]. On the contrary, OSNs expressing M72-OR (the OR not responsive to PEBP1 in in vitro experiments) converge to form glomeruli in similar locations in control and KO mice, corroborating the notion that M72 is activated by a different ligand than PEBP1 [67] (Fig. 6). Altogether, the work by Zamparo and collaborators provided compelling evidence that the axonal ORs are directly involved in guiding OSNs to their target and identified the first putative ligand of a subset of axonal ORs. These findings recapitulate the classic paradigm of expression of guidance cues observed in most systems, including the visual one, where sets of receptors and ligands are expressed in a complementary manner in the projecting sensory axons and in the target areas. In this scenario, the expression of Neuropilin1 and Semaphorin3A [116] and Neuropilin 2 and Semaphorin3F [117] in the OSNs represent an exception.

### The ORs cooperate with several molecular cues to govern OSN axon targeting

The ORs play an instructive role in the formation of the sensory map, but they are not the only guidance cues involved. Several other sets of ligand-receptors contribute, along with the ORs, to direct sensory axons to their glomerular target along distinct axes: antero-posterior (A-P), medio-lateral (M-L), and dorso-ventral (D-V).

The location of glomeruli along the A-P axis is regulated by distinct sets of ligand-receptors, such as the Ephrin-Eph proteins. The large family of Eph tyrosine kinase receptors and their membrane-bound ligands, Ephrins, regulate the generation and maintenance of cellular spatial patterns, and topographic organization, in many tissues [118]. In the OS, sensory neurons were shown to express EphrinA3 and EphrinA5, whereas EphA tyrosine kinase receptors were found to be expressed in the postsynaptic cells of the OB [119–123]. Cutforth in the Axel lab (2003) demonstrated that OSNs expressing different ORs express different levels of EphrinA3 or EphrinA5 in their axons. As a result of this expression pattern, as observed also in previous work [120, 121, 123], glomeruli with high expression level of EphrinA are interleaved with glomeruli in which EphrinA expression is very low. This patchy expression pattern of Ephrin, in contrast to the continuous gradient observed in other sensory systems, such as the visual system, reflects the discrete nature of the glomerular map in the OB. While the expression of the Eph receptors in the postsynaptic cells of the OB, in respect to the Ephrin ligands in OSNs, recapitulates the complementary expression pattern ligand-receptors in sensory afferents and target region (or vice versa) exploited to drive input neurons in specific locations of the target area, in most sensory systems. The role of EphrinA in the formation of the sensory map was unraveled by genetic manipulations of their expression. Deletion of EphrinA3 and EphrinA5 was reported to cause a shift toward the OB posterior pole of glomeruli formed by neurons expressing P2-OR and SR1-OR. Whereas overexpression of EprhinA5 in P2-OR neurons resulted in an anterior shift of the corresponding glomeruli. Altogether, these data demonstrate that EphrinA3 and EphrinA5 play an instructive role in the convergence of OSN axons to form glomeruli along the A-P axis [119]. Other cues involved in the location of glomeruli along the A-P axis are the receptor Neuropilin1 (Nrp1) and its repulsive ligand, Semaphorine3A (Sema3A) (Imai et al. 2006, 2009). These molecules are known to act as axon guidance cues for several neuronal types during development [124–126]. The expression of Nrp1 was found to be correlated to the OR type. Namely, Imai et al. found that different levels of the OR-derived cAMP regulate the transcription of different levels of Nrp1 which exhibits a gradient posterior-high/anterior-low (Imai et al. 2006 but see also [127]. In contrast to the classic model of complementary expression of guidance cues (i.e., receptors - ligands) in neuronal afferents and target area, Imai et al. (2006) found that Nrp1 and its repulsive ligand Sema3A are both expressed in subset of OSNs, in a complementary manner, such that when one is high, the other is low. In addition, they discovered that Nrp1-Sema3A determine not only the projection sites in the target but also the pre-target sorting of axons. In other words, before reaching the OB, the incoming OSNs expressing different ORs, hence different levels of Nrp1-Sema3A, occupy distinct locations within the OSN bundle. Deletion of Nrp1 or Sema3A caused a shift in the glomeruli position along the A-P axis and disrupted the pre-target axon sorting. According to these results, the levels of axon guidance cues present in the axons impact the spatial segregation of sensory afferents even before the target [116, 128]). It is worth noticing that a signature of the molecules contributing to the spatial segregation of sensory afferents along the A-P axis is their highly correlated expression with the OR type.

OSNs expressing the same OR converge to form a glomerulus on the medial and a glomerulus on the lateral side of each OB, resulting in two symmetric maps of isofunctional glomeruli. How is OSN segregation along the L-M axis regulated? Insulin growth factor (IGF) signaling was discovered to organize sensory projections along the M-L axis. IGF family includes two secreted polypeptides ligands, IGF 1 and IGF 2, which bind a common receptor tyrosine kinase, the type 1 IGF receptor (IGF1 R) [129]. IGF signaling is known to regulate cell proliferation and survival, hence body size [129]. Subsequently, it has been discovered that IGF can also modulate axon elongation and targeting, in some brain areas [130, 131]. In the OB, IGF1 is expressed underneath the nerve layer, in proximity of the incoming sensory axons. Interestingly, a medio-lateral gradient of IGF1 was observed in the rostral part of the OB, such that here IGF1 expression is higher on the lateral than on the medial side of the OB. This gradient diminishes and eventually reverses in the caudal part of the OB where IGF expression is higher on the medial than on the lateral side. Consistent with the complementary expression of ligand-receptor cues in sensory axons and target areas, IGF1 R were reported to be expressed in the OSNs. Deletion of IGF1 R results in a dramatic reduction of the OSN innervation of the lateral side of the OB. Furthermore, axons destined to the later side, reroute towards more ventral and medial positions. Null mutation in IGF 1 or IGF 2 did not affect OSN projections, leaving the sensory map unaffected. However, when both IGF 1 and IGF 2 expression was abolished (double KO), the innervation of the lateral aspect of the OB was dramatically reduced. Furthermore, IGF 1 was demonstrated to act as chemoattractant for OSN growth cones, in vitro. Altogether, these data demonstrate that IGF signaling is required for proper innervation of the lateral part of the OB. Lack of IGF signaling causes a dramatic reduction of axon projections on the lateral side of the OB, resulting in a deep disruption of the symmetric maps of homologous glomeruli [132]. How and whether the intrabulbar link between homologous glomeruli is affected by mutations of IGF signaling remains to be investigated.

The location of glomeruli along the D-V axis of the OB is known to be dictated by the position of sensory neurons in the MOE and not by the OR identity. In situ hybridization experiments [73, 74] and tracing experiments [70, 133–135] demonstrated a correspondence between zones in the MOE and zones in the OB. According to this plan, OSNs located in the most dorsal zone of the MOE project to the most dorsal aspect of the OB, while OSNs harbored in the most ventral region of the MOE project ventrally in the OB [70, 133–135]. This pattern of projections could suggest that each zone instructs OSNs to express a set of molecules that direct their axons in corresponding zones of the OB. However, since OR expression is continuous and overlapping across the dorso-medial to ventro-lateral axis of the MOE [70–72], it is more likely that gradient of cues along the D-V axis of the MOE direct OSNs to the corresponding regions in the OB. Two sets of such molecules have been identified: Robo-2-Slit-1 [136–139] and Neuropilin2 (Nrp2)-Semaphorine3F (Sema3F) [117, 138, 140, 141]. Both these sets of molecules are known to guide axons toward their target, in several systems during development [142, 143].

Robo-2, a receptor for Slit chemorepellents, is expressed in a high dorso-medial and low ventro-lateral gradient in the OB. Null mutation for Robo-2 causes a rerouting of axons from the dorsal area toward the ventral aspect of OB, indicating that Robo-2 is required for dorsal targeting. Furthermore, ablation of Slit expression caused defects similar to the one observed in Robo-2 mutants. These results indicate that Slit-1-Robo2 favor the dorsal targeting of OSNs [137, 144]. In addition, it has been shown that the targeting of P2-OR and MOR 28-OR neurons, which innervate two distinct regions of the medial aspect of the OB, is altered in Slits (Slit-1 and Slit-3) - Robo-2 mutants, indicating that these cues are required also for proper innervation of the ventral areas of the OB [136]. Sakano group found that also Robo 1, expressed in the ensheathing cells, but not in OSNs, could contribute to the D-V targeting [145]. Worth noticing, another cue, Nrp2, is expressed in a dorso-ventral gradient in the MOE, suggesting that it can also contribute to spatial segregation of sensory afferents along the D-V axis. Evidence indicated that Nrp2, an axon receptor, and Sema3F—its repulsive ligand, are involved in pruning the overshooting of sensory axons into the external plexiform layer [138, 140, 141]. Takeuchi et al. (2010) found that Nrp2 and Sema3F are both expressed in OSNs, although in a complementary manner. According to this model, Sema3F is secreted by early arriving OSNs that target the antero-dorsal region of the OB to repel OSN axons that arrive later. The sequential innervation of the OB and the complementary expression of Nrp2 Sema3F in the same neurons contribute in sensory axon segregation along the D-V axis [117, 128].

The formation of the olfactory map is a multi-step process in which a coarse spatial segregation of sensory afferents is then followed by the coalescence of like-axons to form distinct glomeruli. The latter phase is likely to be mediated by cell-adhesion molecules. To understand the mechanism underlying glomeruli formation, Serizawa et al. (2006) searched for molecules whose expression was correlated with the expressed ORs. They found that the adhesion molecules Kirrel2/Kirrel 3 and Ephrin-A ligands and EphA were expressed in subsets of OSNs, in a complementary manner. Abolishing OR-evoked activity in CNG KO mice lead to downregulation of Kirrel2 and EphA5 and upregulation of Kirrel 3 and Ephrin-A5. As far as the impact of these molecules on the sensory map, overexpression of these genes in half of OSNs expressing specific OR results in duplicated glomeruli [146].

BIG-2-contactin 4 is an axonal glycoprotein belonging to the superfamily of the immunoglobulin, whose expression is highly correlated with the OR identity [147, 148]. The patchy pattern of expression of BIG2 results in a mosaic of glomeruli with distinct levels of BIG2. This patter resembles, but does not overlap, with the expression of Ephrins and Kirrels. In mice carrying a null mutation in BIG-2, OSNs expressing given ORs targeted multiple glomeruli in aberrant locations, indicating that BIG-2 is required for proper glomeruli formation [147]. More recently, it has been reported that the cluster of cell surface proteins Protocadherins (Pcdh), encoded by three linked genes Pcdh α, β, and γ, are all required for OSN axonal convergence. Notably, deletion of one of the genes of the Pcdh cluster minimally affects the formation of glomeruli, while deleting the three clusters results in severe defects in axonal arborization that prevents glomeruli formation. The three clusters of Pcdh appear required to confer to OSN axons expressing different OR sufficient diversity to coalesce in distinct glomeruli [149]. Altogether, these data indicate that several guidance cues cooperate with the OR to direct sensory axons to their targets (Table 1).

**Table 1:** Molecular cues which contribute in directing olfactory sensory neuron projections to the olfactory bulb

Antero-posterior axis	Nrp1- Sema3A, Plexin1 EphrinA, EphrinA3 - Eph-A5, EphA3
Dorso-ventral axis	Slits-Robo2, Robo1 Nrp2-Sema3F
Medio-lateral axis	IGF1, IGF2 - IGF1R
Adhesion molecules	Kirrel2/Kirrel3 BIG2 Pcdh

### Odorant receptors and neuronal activity

The development of topographic maps is regulated by a complex interplay between neuronal activity and molecular cues. Despite the central role of the ORs in defining the olfactory topography, odorant-evoked activity does not significantly affect the formation of the sensory map, as demonstrated by several transgenic line of mice carrying genetic mutations of key elements of the OR transduction pathway, such as CNG channels [150–152] and Golf [153]. Different results were observed upon the ablation of adenylyl cyclase III (ACIII) [90–92]. This manipulation prevents the synthesis of cAMP upon OR activation. In this line of anosmic mice, the coalescence of sensory axons to form glomeruli was deeply perturbed. These results indicate that odorant-evoked activity is dispensable for the sensory map formation and corroborate the role of the OR-derived cAMP in the coalescence of like-axons to form glomeruli [34, 89, 116, 154, 155]. Indeed, among the genetic manipulations that eliminate odorant-evoked activity, only the ablation of ACIII perturbed the formation of glomeruli. Mutations in genes encoding CNG channels and Golf abolish responses to odorants but maintain cAMP synthesis, allowing a normal development of the sensory map.

In contrast, spontaneous afferent discharge of OSNs plays a critical role in the refinement and maintenance of the sensory projections, although it does not have an instructive role in the formation of the sensory map [156–158]. Noteworthy, in OSNs, the ORs dictate not only the response profile to odorants but also the basal activity. OSNs expressing different ORs exhibit different spontaneous firing rates [45, 46]. In addition, OSNs expressing an inactive mutant OR (DRY mutant, see above and Imai, 2006) completely lack spontaneous firing, despite being able to generate action potentials in response to current injection [45]. Altogether, these data indicate that the spontaneous activation of the ORs is the origin the spontaneous firing of OSNs.

To ascertain whether the spontaneous OR activation could be the origin of the OR-derived cAMP, which in turn regulate axon guidance cues, Nakashima et al. (2013) generated transgenic line of mice expressing the beta-adrenergic receptors (B-AR) under the OR promoters. They devised this approach since the initial experiments with ORs were not successful due to the large number of different ORs and the lack of crystal structure of the ORs, which make difficult to screen the OR spontaneous activity. The B-AR is known to exhibit ligand independent activity, resulting in a basal level of cAMP (see above). Nakashima et al. (2013) found that mutants with altered level of basal activity changed the transcription levels of Neuropilin1 and Plexin1, which in turn impaired the position of glomeruli along the A-P axis. The expression of adhesion molecules such as Kirrel2 and Kirrel3 was not affected [48]. In a more recent work, Nakashima et al. (2019) [159] found that OSNs expressing different ORs exhibit different spontaneous profile of Ca2+ transients, confirming results obtained in previous works, related to the spontaneous firing rate of OSNs [45, 46]. To ascertain how the spontaneous activity of OSNs could affect the expression of axon guidance cues, they exploited optogenetic stimulation of OSNs. They found that short burst of activity regulated Kirrel2 expression, while prolonged stimulation induced the expression of Semaphorin7A and protocadeherin 10 [154, 159]. Therefore, whether spontaneous afferent activity plays an instructive role [48, 159] or a permissive role [156, 158] remains to be clarified.

## Conclusions—perspectives

In this review, we provided an overview of the critical steps that unraveled the role of the ORs in the formation of the olfactory map. The specificity of connections in the OB results in a discrete topographic organization which hinges on the OR identity. Evidence indicates that the OR is not only the feature which shapes the OB neuronal wiring into glomerular units but also the molecule that directs OSN axons in specific locations in the OB. However, although the ORs play an instructive role in OSNs targeting, they are not the only determinant.

The current view is that the ORs exert their action via the OR-derived cAMP (Imai et al., 2006), which in turn regulates the expression of other guidance cues. As regards the origin of cAMP, it has been reported that agonist-independent activation of the OR produces basal levels of cAMP. In this model, OSNs expressing a given OR exhibit a specific level of cAMP and basal firing activity that in turn regulate the expression of specific levels of guidance cues [48, 154, 159]. Alternatively, the activation of the axonal ORs by cues elaborated in the OB may represent the origin of the OR-derived cAMP [86]. The axonal OR-derived cAMP is likely to act locally at the axon terminal to steer the growth cone and at the nucleus, via PKA activation, to regulate the expression of other guidance cues. Recent findings unraveled the signaling pathway coupled to the axonal ORs [96, 97] and identified the first putative ligand of a subset of axonal ORs [67], corroborating this hypothesis. Such a model does not entail the existence of a thousand different cues in the OB, one for each OR type, but proposes that a few cues elaborated in the OB bind, with different affinities, distinct subsets of ORs. In line with hypothesis, PEBP1, the first putative ligand of a distinct subpopulation of axonal ORs, was shown to activate several axonal ORs although with different affinity [67]. In this context, the specificity of sensory projections is achieved by the different affinity of ORs for the OB cues. In addition, the expression at the axon terminal of a unique combination of axon guidance cues along with the ORs contribute to direct OSN axons to specific glomerular targets. The coalescence of like-axons to form glomeruli is then regulated by adhesion molecules and refined by afferent spontaneous activity.

Although the identification of the first putative ligand of the axonal ORs provides novel insights on their role in the OB topography, it also opens several questions. Namely, the identity of other putative OR ligands remains to be deciphered. Whether the cues elaborated in the OB drive not only sensory axons to their target but also regulate the link between homologous glomeruli remain to be investigated. Since external tufted cells link glomeruli expressing the same ORs, it is tempting to speculate that molecules whose expression is regulated by the OR guide also the projections of the external tufted cells. Whether these molecular cues are the same or different in respect to the ones which guide OSN axons to their glomerular target, remain to be investigated. Addressing these open questions is critical to have a complete picture of the cues involved in directing OSNs and external tufted cells to their targets and distill a model that describes the mechanism underlying the OB topography.

## Data Availability

Not applicable
